# Environmental drivers of vertigo

**DOI:** 10.1265/ehpm.25-00257

**Published:** 2026-02-17

**Authors:** Benyamin M Kaminer, Naama Schor, Tali Shorer, Itay Pansky, Victor Novack, Lena Novack

**Affiliations:** 1 Department of Otolaryngology-Head & Neck Surgery, Soroka University Medical Center, Beer-Sheva, Israel; 2 School of Public Health, Faculty of Health Sciences, Ben-Gurion University of the Negev, Beer-Sheva, Israel; 3 Negev Environmental Health Research Institute, Soroka Clinical Research Center, Soroka University Medical Center, Beer-Sheva, Israel; 4 Soroka University Medical Center, Beer-Sheva, Israel; 5 Internal Medicine Department, Soroka University Medical Center, Beer-Sheva, Israel; 6 The Goldman Sonnenfeldt School of Sustainability and Climate Change

**Keywords:** Vertigo, Environmental, Epidemiology, Barometric pressure, Heatwave, Humidity, Hypertension

## Abstract

**Background:**

Vertigo is characterized by the illusion of motion, typically described as a spinning sensation. Seasonal variations in vertigo incidence, particularly in Meniere’s disease and Benign Paroxysmal Positional Vertigo, have been reported, though underlying mechanisms remain unclear.

**Objective:**

This study investigates key environmental drivers by analyzing seasonal patterns of vertigo while accounting for temperature, barometric pressure, humidity, and viral infections in Israel’s semi-arid Negev desert. We hypothesized that environmental conditions are associated with the onset of vertigo, and that specific meteorological factors contribute to its seasonal variation.

**Methods:**

A case-crossover study was conducted using data from 9,382 patients diagnosed with vertigo at Soroka Medical Center (2014–2019). Environmental exposure data were collected from local meteorological station, and viral infection trends were analyzed.

**Results:**

Exposure to barometric pressure above the 90th percentile for two consecutive days was linked to a 21% increase in vertigo risk (p = 0.024), rising to 40% (p = 0.073) among hypertensive patients. In spring, exposure to extreme dry heat (>2 days) showed a trend toward increased vertigo risk. Similarly, vertigo onset was likely to be triggered by heatwaves stress and extremely humid conditions in fall although none reached a statistical significance.

**Conclusions:**

This study suggests that atmospheric conditions, particularly high barometric pressure, may influence vertigo onset. While not all associations were statistically significant, observed trends highlight the potential role of environmental factors in vertigo and warrant further research.

**Supplementary information:**

The online version contains supplementary material available at https://doi.org/10.1265/ehpm.25-00257.

## 1. Introduction

Vertigo, a subtype of dizziness, is characterized by the illusion of motion, typically described as a spinning sensation of oneself or the surroundings [1]. Vertigo occurs to approximately 2–5% of the general population [2]. The etiologies of vertigo are diverse, broadly categorized into peripheral and central causes. Peripheral vertigo, the most common type, arises from disorders affecting the inner ear or vestibular nerve, such as Benign Paroxysmal Positional Vertigo (BPPV), Acute Vestibulopathy and Meniere’s Disease [3, 4]. Trauma, infection, and other associated inner ear pathologies are among the known associated causes for these pathologies [5–7].

Central vertigo, less common but potentially more serious, results from dysfunctions in the central nervous system, including conditions like migraines, strokes, or multiple sclerosis [8].

Several studies have investigated the seasonality of vertigo, reporting varying seasonal patterns. Lai et al. described seasonal uneven distribution of vertigo among residents of Taiwan. The researchers found that Vertigo was most common in winter (27.1%) and spring (26.3%) [9]. In contrast, Kim et al. found that Ménière’s disease in a South Korean population occurred more frequently in summer and autumn [10]. The group led by Korpon reported not only seasonality pattern in the disease onset, but also a positive correlation of BPPV onset with barometric pressure [11]. Their findings align with those of Gürkov and colleagues, who likewise observed an influence of barometric pressure on vertigo [12]. However, the effects of barometric pressure on vertigo and on human physiology in general have been scarcely investigated, and existing studies have primarily focused on high-altitude conditions rather than atmospheric pressure fluctuations at ground level.

The underlying mechanisms behind the seasonal distribution of vertigo remain unclear. While opposing trends reported by different groups can be attributed to geographical and meteorological variation in the study regions, the specific seasonal factors triggering the onset of the disease are unknown. Characteristics like temperature, humidity, solar radiation, barometric pressure or viral surges, may all potentially contribute to the vertigo peaks described in literature. Viral infections in particular, have been recognized as triggers of vestibular disorders and vertigo through mechanisms of inner-ear inflammation and vestibular nerve irritation. Recent studies indicate that neurotopic and respiratory viruses, such as herpes simplex virus, SARS-Cov-2, and cytomegalovirus, can induce vestibular neuritis or labyrinthis, leading to acute vertigo episodes [13, 14]. The variability shown highlights the need for further research to clarify the environmental and demographic factors contributing to the seasonality of vertigo. Hence, this analysis aims to identify the key drivers of the condition by examining the seasonal variation in vertigo cases while accounting for temperature, barometric pressure levels, relative humidity and viral infections in population. To date, few epidemiological studies have examined the combined effects of these environmental variables on vertigo, barometric pressure in particular. Notably, desert and semi-arid environments—which cover a substantial and expanding portion of the Earth’s surface—have rarely been investigated in this context. Our study population resides in the semi-arid Negev desert area located in the southern part of Israel, an area characterized by high temperatures during summer, contrasting low temperatures at night and low humidity. We hypothesized that environmental conditions are associated with the onset of vertigo, and that extreme semi-arid conditions of the study area would amplify that possible impact.

## 2. Methods

### 2.1 Study design

We conducted a population-based study using the data on patients admitted to the Soroka University Medical Center (SUMC) between 2014–2019. SUMC is the only hospital providing tertiary care to the population comprising close to 1 million residents of the Southern Israel. In that sense, all cases with vertigo-like symptoms represent unbiased population-based rates of disease in the Southern Israel.

Our study included all patients aged 18 and above, admitted to the emergency department (ED) or hospitalized with a primary diagnosis of vertigo, as identified by the International Classification of Diseases, Ninth Revision (ICD9) codes 438.85, 386.XX, and 780.4 (Table S1). Patient data collected included basic demographics and extensive clinical information. We used a time-stratified case-crossover design case crossover (CCO) design. Specifically, to every case of vertigo we matched up to 5 control days on the same day of the week as the index case and within the same month, to avoid imbalance due to seasonal trends. This strategy is a standard approach in case-crossover studies to control for confounding by seasonality and day-of-week. Essentially, time is divided into strata defined by year-month and weekday, and each case is compared to referent days from the same stratum. By using this time-stratified referent selection, we avoid bias from long-term time trends or seasonal variations because each case and its controls share the same background time context [15, 16]. Subjects with repeated vertigo events were excluded from the analysis, to avoid misclassification of exposure in temporarily adjacent cases. We further compared exposure prior to disease onset with exposure on control days without events.

### 2.2 Environmental exposure assessment

Exposure was assessed using a meteorological monitoring station managed by the Israel Meteorological Services and located in the center of Beer-Sheba city, close by the SUMC. The main population of SUMC patients reside primarily in the city and Beer-Sheba suburbs within the radius of 20 km of the city center. We therefore assumed that the meteorological estimates accurately represented the exposure levels of the study population, although we acknowledge that data from a single station may not fully capture microclimatic variability across the wider Negev region. The monitored values used in the study included temperature, relative humidity and barometric pressure measured every 3 hours.

### 2.3 Exposure to viral morbidity

Exposure to viral infections in the population was estimated based on the national database of the Clalit HMO serving approximately 55% of the population in Israel. As these data reflect national trends rather than region-specific infection rates, some degree of local exposure misclassification cannot be ruled out. We retrieved information on the daily overall count of PCR-confirmed viral infections within Clalit members. The list of viruses included Adenovirus, Influenza A, Influenza B, Enterovirus, Rhinovirus, Respiratory Syncytial Virus (RSV) and Human Metapneumovirus (HMPV). Epidemic periods for influenza A, influenza B, and RSV were identified during winter months when their daily counts exceeded the median count.

### 2.4 Data analysis

Continuous variables were summarized using mean and standard deviation (SD) or median along with minimal and maximal values, while categorical variables were described as percentages and counts.

Seasonality of the monthly series of vertigo cases was tested for annual and semi-annual patterns using sine and cosine harmonic terms in a Poisson regression model.

The CCO design used in the study provided an optimal adjustment for individual confounding factors remaining stable over time. To explore the impact of environmental exposures, we expressed meteorological measurements using two different approaches, the distributed lag nonlinear modeling (dlnm) [17, 18] and a dichotomized version of exposure values. DLNM is the modeling approach that enables the simultaneous estimation of both the exposure-response relationship and the lag-response relationship, providing a framework for capturing delayed and non-linear effects of temperature. To model the exposure, we created an exposure matrix that included the daily average exposure along with lagged values for up to seven days. The crossbasis matrix incorporated both the exposure-response and lag-response dimensions. We specified a natural cubic spline function with three degrees of freedom for the exposure-response relationship, and the same for the lag dimension of delayed effects of exposure. For the exposure-response dimension, 3 degrees of freedom corresponds to a default one knot placed at the median, ensuring the exposure–response curve could capture non-linear effects (e.g. a possible U-shape) without overfitting. Similarly, for the relatively short lag structure of up to 7 days, using df = 3 allowed for one internal knot over the lag period that could capture a non-linear change in effect over time, while keeping the lag structure parsimonious.

We dichotomized exposure values to examine the impact of exposure to extreme levels of temperature, humidity and barometric pressure, defined by either 90^th^ or 10^th^ percentile of the global distribution of measurements for the entire study period. The 90th percentile was selected a priori to capture extreme exposure levels, representing the upper 10% most likely to trigger vertigo through physiological stress. This threshold aligns with common environmental-epidemiology practice, where heat-related studies often define “extreme” conditions using the 90th–95th percentile. Heat waves were defined as extreme heat (above 90^th^ percentile) maintained for at least 2 consecutive days prior to the event or its control day. The wave of extreme levels of humidity and BP levels were defined in a similar manner.

Further, we used conditional logistic regression equation to estimate the Odds Ratio (OR) of exposure to environmental factors and vertigo onset. We adjusted all meteorological states, i.e. temperature, RH and BP, to each other, in all modeling analyses estimating the effect.

Statistical significance was claimed at the level of 5%. The statistical analysis was performed using SAS9.4 and R Studio (R version 4.4.1).

## 3. Results

A total of 12,086 patients were treated at the hospital for vertigo symptoms. Among them, 9,382 patients (77.6%) received treatment only once and were included in the study. Of these, 5,953 were diagnosed by a general diagnosis of “Dizziness and giddiness” (ICD9 780.40) and the rest 3429 patients received a more specific diagnosis of vertigo, i.e. 438.85 or 386.XX. Out of the latter group, 1253 patients were also seen by an otolaryngologist (ENT) specialist. Most cases had 4 control days within the same month (90.1%), 1.5% of them had 3 control days and 8.0% had 5 control days. The patients were 53.4 years old on average, 57.3% of them were female and 78.6% were of Jewish ethnicity (the rest being of Bedouin-Arab ethnicity) (Table 1). Close to 20% of the patients had a chronic diagnosis of hypertension (HTN) and 14.1% were diagnosed with diabetes mellitus (DM). Approximately 13% of the patients were hospitalized due to vertigo symptoms. There were no apparent differences between the patients with a general diagnosis and specific diagnosis of vertigo.

**Table 1:** Demographic and clinical characteristics of the study population

**Patients’ Characteristics**	**Dizziness and** **giddiness (Vertigo)** **(N = 5953)**	**Certain Vertigo** **(N = 3429)**		**Total (N = 9382)**

		**Vertigo, specific** **diagnosis** **(N = 2176)**	**Vertigo, specific** **diagnosis** **confirmed by ENT** **(N = 1253)**	
**Demographical characteristics**				
Age, years				
Mean ± SD (n)	53.0 ± 20.2 (5953)	54.5 ± 18.0 (2176)	53.7 ± 16.5 (1253)	53.4 ± 19.2 (9382)
Median	55.0	57.0	55.0	55.0
Min; Max	19.0; 111	19.0; 94.0	19.0; 111.0	19.0; 111.0
Male gender, % (n/N)	43.7 (2602/5952)	42.1 (917/2176)	38.9 (487/1253)	42.7 (4006/9381)
Ethnicity, % (n/N)				
Jewish	76.6 (4556/5947)	80.2 (1741/2172)	85.6 (1072/1252)	78.6 (7369/9371)
Bedouin Arab	23.4 (1391/5947)	19.2 (431/2172)	13.4 (180/1252)	21.4 (2002/9371)
Immigrants, % (n/N)	43.9 (2612/5953)	45.8 (996/2176)	45.8 (574/1253)	44.6 (4182/9382)
Immigrated within 5 years prior to the event, % (n/N)	1.5 (40/2612)	0.9 (9/996)	0.7 (7/574)	1.3 (53/4182)
**Medical History**				
Hypertension, % (n/N)	19.3 (1148/5953)	20.8 (452/2176)	18.4 (230/1253)	19.5 (9382)
Diabetes Mellitus, % (n/N)	14.4 (858/5953)	14.4 (313/2176)	12.2 (153/1253)	14.1 (1324/9382)
Smoking history, % (n/N)	35.4 (1729/4879)	34.3 (597/1739)	30.7 (305/995)	34.6 (2631/7613)
**Index ED visit for Vertigo**				
Hospitalized, % (n/N)	14.0 (834/5953)	8.8 (191/2176)	15.9 (199/1253)	13.1 (1224/9382)
Length of stay, days				
Mean ± SD (n)	4.8 ± 6.3 (834)	4.8 ± 7.0 (191)	4.1 ± 4.0 (199)	4.7 ± 6.1 (1224)
Median	3.0	3.0	3.0	3.0
Min; Max	1.0; 69.0	1.0; 72.0	1.0; 42.0	1.0; 72.0
Fever (Body temp ≥37.0 °C), % (n/N)	16.2 (773/4780)	13.1 (217/1612)	10.2 (114/1115)	14.7 (1104/7507)
Pulse				
Mean ± SD (n)	81.7 ± 15.5 (5385)	79.5 ± 14.8 (1834)	79.7 ± 14.1 (1250)	80.9 ± 15.2 (8467)
Median	80.0	78.0	78.0	79.0
Min; Max	36.5; 166.0	47.0; 150.0	36.7; 146.0	36.5; 166.0
Systolic Blood Pressure				
Mean ± SD (n)	135.2 ± 20.9 (5397)	136.9 ± 20.0 (1836)	135.7 ± 19.9 (1251)	135.6 ± 20.6 (8484)
Median	134.0	135.5	134.0	134.0
Min; Max	59.5; 216.5	69.5; 213.5	56.5; 205.5	56.5; 216.5
Diastolic Blood Pressure				
Mean ± SD (n)	75.8 ± 12.1 (5396)	76.6 ± 12.1 (1836)	76.6 ± 11.6 (1251)	76.1 ± 12.1 (8483)
Median	75.0	76.0	76.5	76.0
Min; Max	27.0; 151.0	34.0; 135.0	34.5; 151.5	27.0; 151.5
Systolic Blood Pressure, % (n/N)				
Below 90	1.6 (87/5397)	1.1 (20/1836)	1.5 (19/1251)	1.5 (126/8484)
Above 140	46.0 (2481/5397)	48.5 (890/1836)	49.8 (623/1251)	47.1 (3994/8484)
Saturation <95, % (n/N)	12.9 (637/4936)	12.8 (212/1655)	11.9 (136/1148)	12.7 (985/7739)

The temperatures were the highest in summer with a daily average reaching 26 °C and the lowest in winter, with daily average around 13 °C (Table 2). Heat waves (2+ consecutive days of temperature >27.9 °C) occurred 8.2 times a year, in all, 49 times during the study period.

**Table 2:** Meteorological conditions in Southern Israel during the study period (2014–2019)

**Meteorological** **conditions**	**Entire study** **period**	**Winter** **Dec7–Mar30**	**Spring** **Mar31–May30**	**Summer** **May31–Sep22**	**Fall** **Sep23–Dec6**
Temperature, °C					
Mean ± SD	20.5 ± 6.2	13.5 ± 3.2	21.5 ± 4.1	26.9 ± 1.7	20.5 ± 3.7
Median	21.2	13.1	21.1	27.1	20.8
10^th^ percentile	11.8	9.9	16.4	24.8	15.3
90^th^ percentile	27.9	18.0	27.0	28.8	25.0
Min; Max	4.8; 33.9	4.8; 26.8	11.8; 33.9	21.5; 32.4	8.6; 29.6

Relative Humidity, %					
Mean ± SD	61.8 ± 13.7	66.3 ± 14.3	53.5 ± 15.1	61.2 ± 8.4	62.7 ± 14.8
Median	64.1	68.6	57.3	63.6	66.4
10^th^ percentile	42.5	45.4	30.9	50.2	42.6
90^th^ percentile	76.9	83.1	70.5	68.3	77.0
Min; Max	7.4; 99.9	20.3; 99.9	12.0; 82.0	18.6; 78.4	7.4; 89.4

Barometric Pressure, hPa					
Mean ± SD	981.4 ± 4.3	984.6 ± 4.2	980.6 ± 3.0	977.6 ± 2.2	983.0 ± 2.7
Median	981.0	984.7	980.8	977.5	982.7
10^th^ percentile	976.0	979.1	976.7	974.8	979.9
90^th^ percentile	987.5	989.9	984.2	980.5	986.6
Min; Max	970.5; 996.2	970.5; 996.2	973.1; 991.2	971.5; 984.6	974.9; 995.4

**Waves of extreme conditions during the study period**		
Heat above 90^th^ percentile	Number of days or waves	77
	Length of waves, days (Min; Max)	1; 23
	Number of waves 2+ days long	49 (8.2 per year) 0 in winter, 8 in spring, 39 in summer, 2 in fall

Humidity below 10^th^ percentile	Number of days or waves	121
	Length of waves, days (Min; Max)	1; 9
	Number of waves 2+ days long	55 (9.2 per year) 12 in winter, 22 in spring, 11 in summer, 10 in fall

Humidity above 90^th^ percentile	Number of days or waves	99
	Length of waves, days (Min; Max)	1; 9
	Number of waves 2+ days long	52 (8.7 per year) 39 in winter, 1 in spring, 0 in summer, 12 in fall

Barometric pressure below 10^th^ percentile	Number of days or waves	89
	Length of waves, days (Min; Max)	1; 9
	Number of waves 2+ days long	52 (8.7 per year) 2 in winter, 6 in spring, 44 in summer, 0 in fall

Barometric pressure above 90^th^ percentile	Number of days or waves	79
	Length of waves, days (Min; Max)	1; 11
	Number of waves 2+ days long	50 (8.3 per year) 42 in winter, 3 in spring, 0 in summer, 5 in fall

Heat above 90^th^ percentile and humidity below 10^th^	Number of days or waves during the study period	37
	Length of waves, days (Min; Max)	1; 4
	Number of waves 2+ days long	12 (2 per year) 0 in winter, 6 in spring, 6 in summer, 0 in fall

Heat above 90^th^ percentile and humidity above 90^th^	Number of days or waves during the study period	0
	Length of waves, days (Min; Max)	
	Number of waves 2+ days long	

RH was the highest in winter, 66.3%, and lowest in spring, 53.5%. The RH 10^th^ percentile was 42.5%, however the 90^th^ percentile was only 76.9%. The peak of BP was in winter, 984.6 hPa, and the lowest in summer, 977.6 hPa. Waves of extremely high BP (>90^th^ percentile 987.5 hPa) occurred 50 times during the study period (8.3 times a year).

**Fig. 1:**
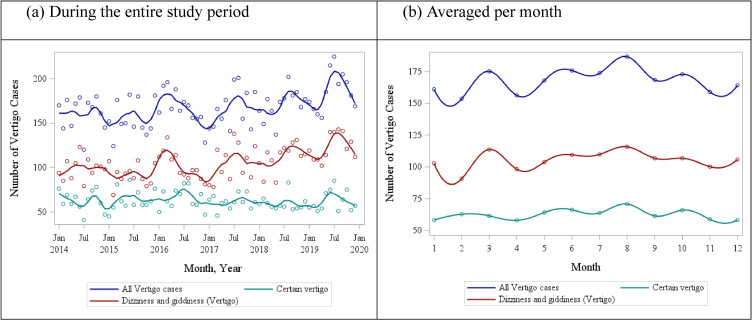
Distribution of vertigo cases, by type of vertigo diagnosis

**Fig. 2:**
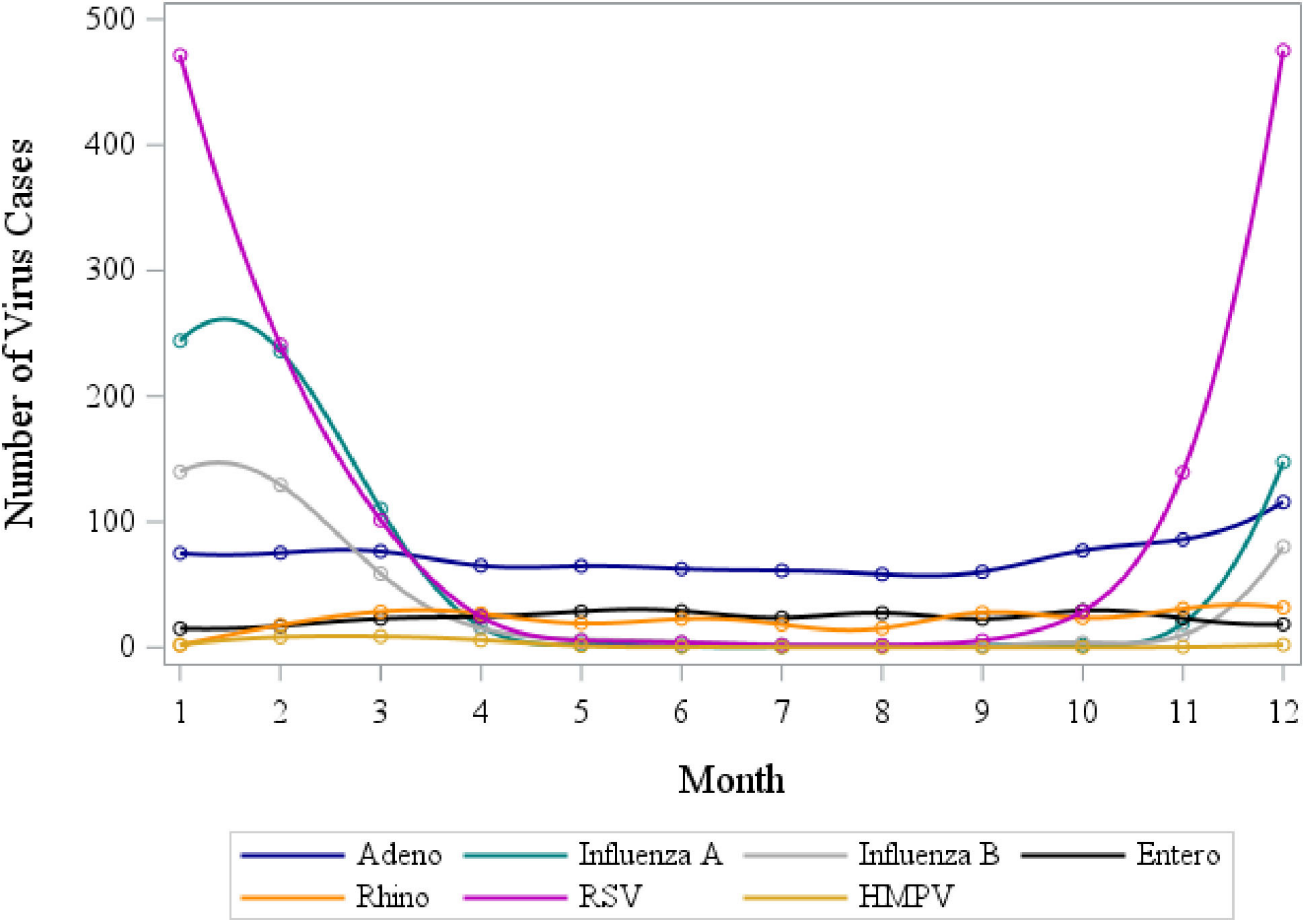
Distribution of infection diseases in Israel, averaged per month

Judging by significance of harmonic terms, the distribution of all vertigo cases followed a strong annual, but not semi-annual, pattern for all types of vertigo definitions (data not shown). Vertigo cases peaked around the month of July reaching 186 cases on average per month and decreased to a minimum of 153 cases in the colder month of February (Fig. 1a, b). Temporal distribution of viral cases confirmed by PCR was different from that of the vertigo cases, with Adenovirus, Rhinovirus, Enterovirus and HMPV remaining generally constant throughout the year and Influenza A, Influenza B and RSV peaking in winter months (Fig. 2).

Further we explored an association of the disease onset and meteorological states. This assessment was performed on the patients defined by a more specific diagnosis of (438.85 or 386.XX), in all 3429 patients.

In the explorative analysis of the association using distributed lag analysis, high temperatures showed a non-significant but suggestive association with vertigo onset (Fig. S1). The effect of humidity varied across different RH values, with lower RH conditions showing a non-significant but suggestive trend for an adverse effect.

The analysis of waves of extreme weather conditions is presented in Table 3. It did not indicate a statistically significant association of the disease onset with heatwaves, while the effect for the year-round period was equal OR = 1.15 (p-value = 0.114) (Table 3). Similarly, extreme humidity weather conditions were not associated with vertigo for both extremely dry and extremely humid conditions, except for a fall where a wave of at least two consecutive humid days showed a suggestive association with vertigo onset (OR = 1.47, p-value = 0.055). The combination of extreme dry conditions lasting 2 or more consecutive days and heatwaves appeared to be suggestively linked to the onset of vertigo; however, the interaction term only showed a trend in spring (OR = 5.39, p-value = 0.115).

**Table 3:** Association between meteorological states and vertigo onset

**Meteorological state^2^**	**All year-round**	**Winter** **Dec7–Mar30**	**Spring** **Mar31–May30**	**Summer** **May31–Sep22**	**Fall** **Sep23–Dec6**
**Heatwave^3^**					
OR^1^ (95%CI)	1.15 (0.97; 1.38)	No obs	1.33 (0.82; 2.16)	1.13 (0.93; 1.37)	0.49 (0.11; 2.27)
p-value	0.114		0.245	0.236	0.360
**Humidity**					
Dry wave^4^					
OR^1^ (95%CI)	0.94 (0.77; 1.14)	1.07 (0.68; 1.69)	1.06 (0.75; 1.50)	0.88 (0.53; 1.45)	0.92 (0.62; 1.35)
p-value	0.533	0.770	0.746	0.608	0.654
Humid wave^4^					
OR^1^ (95%CI)	1.07 (0.90; 1.27)	0.98 (0.80; 1.20)	1.58 (0.40; 6.18)	No obs	1.47 (0.99; 2.19)
p-value	0.444	0.858	0.511		0.055
**Joint effect of heatwave and dry wave**					
Heatwave					
OR^1^ (95%CI)	1.10 (0.91; 1.33)	No obs	0.29 (0.04; 2.23)	1.11 (0.91; 1.35)	No obs
p-value	0.342		0.236	0.309	
Dry wave					
OR^1^ (95%CI)	0.87 (0.71; 1.08)	No obs	0.80 (0.56; 1.15)	0.71 (0.33; 1.51)	No obs
p-value	0.208		0.226	0.373	
Heatwave x Dry wave					
OR^1^ (95%CI)	1.35 (0.86; 2.11)	No obs	5.39 (0.66; 43.78)	1.57 (0.60; 4.12)	No obs
p-value	0.189		0.115	0.361	
**Barometric Pressure (BP)**					
Low BP wave^5^					
OR^1^ (95%CI)	1.06 (0.89; 1.27)	No obs	1.07 (0.89; 1.29)	1.04 (0.84; 1.30)	No obs
p-value	0.507		0.581	0.712	
High BP wave^5^					
OR^1^ (95%CI)	1.21 (1.03; 1.43)	1.13 (0.94; 1.37)	3.52 (1.28; 9.72)	No obs	1.35 (0.78; 2.35)
p-value	0.024	0.188	0.015		0.286

^1^Risk estimates of waves of extreme levels of one meteorological condition, are adjusted to the levels of the two other conditions on the day of vertigo onset or its control. For instance, heatwaves are adjusted to the average levels of RH and BP on the day of the event or its control.

^2^An interaction between waves of extreme humid weather conditions and heat were not explored as these two conditions did not occur simultaneously in the study area.

^3^A heatwave was defined as ≥2 consecutive days with mean temperature above the 90th percentile (27.9 °C) for the entire study period.

^4^A dry wave and humid wave were defined as ≥2 consecutive days with relative humidity below the 10^th^ percentile (42.5%) or above the 90^th^ percentile (76.9%), respectively.

High and low barometric pressure (BP) waves were defined as ≥2 consecutive days with mean BP above the 90th percentile (987.5 hPa) or below the 10th percentile (976.0 hPa).

High barometric pressure exhibited a stable adverse effect across all seasons, with OR = 1.21 (p-value = 0.02) for the year-round analysis and OR = 3.52 (p-value = 0.015) in spring.

**Fig. 3:**
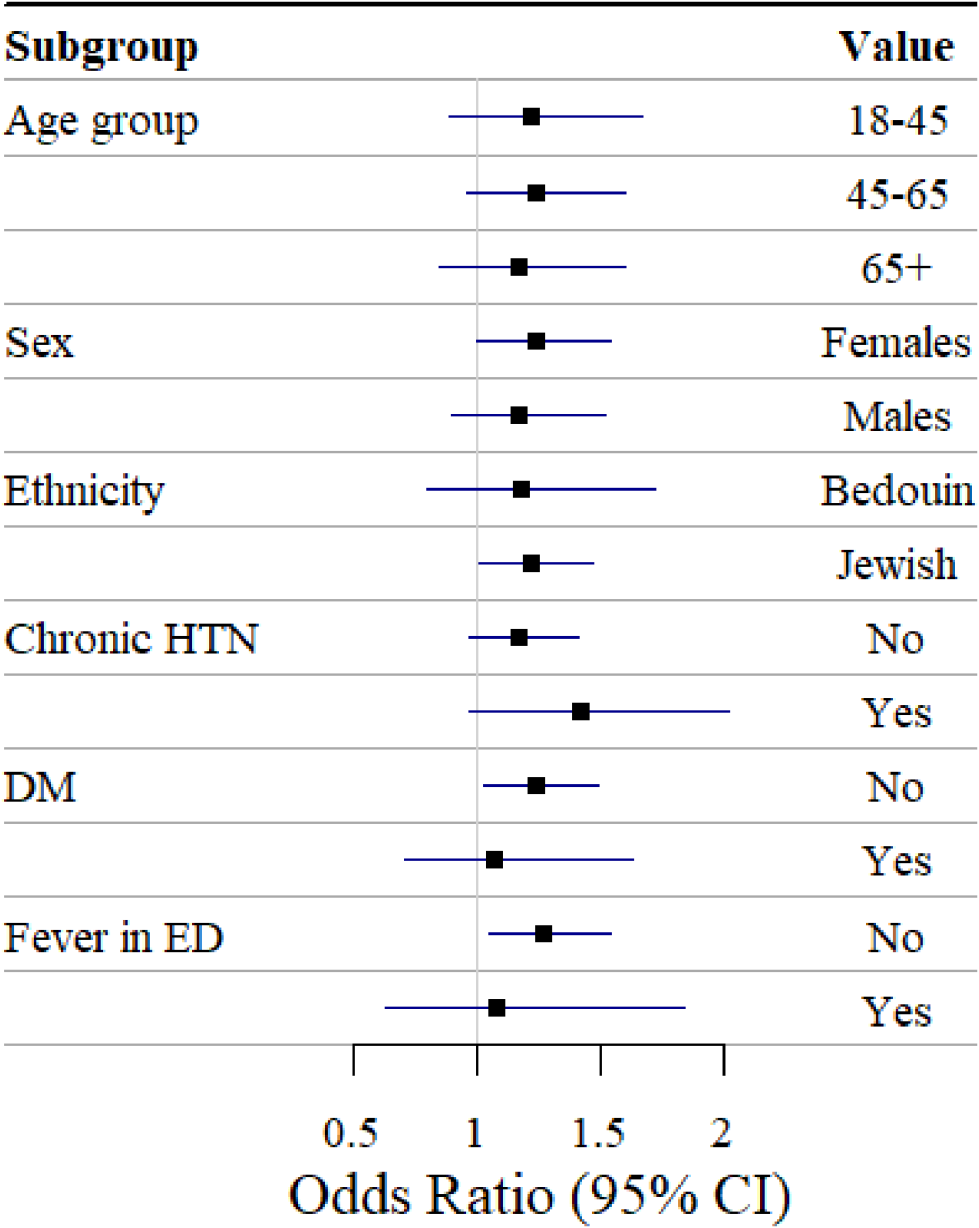
Association between a wave of extremely high barometric pressure and vertigo onset. Results of subgroup analysis. ^1^All effect estimates are adjusted to temperature and RH on the day of the event onset or its control.

The subgroup analysis showed patients diagnosed with chronic hypertension to be at the highest risk of vertigo (OR = 1.42, 95%CI: 0.98; 1.45) (Fig. 3 and Table S2). Additionally, patients admitted to ED without fever were at higher risk of being diagnosed with vertigo (OR = 1.26, 95%CI: 1.04; 1.53).

## 4. Discussion

Based on our analysis, simultaneous exposure to waves of hot and dry weather showed a non-significant but suggestive association with vertigo onset. For instance, a combination of low humidity and high ambient temperature over at least 2 consecutive days presented with an OR of 5.39 for a vertigo in spring, as compared to colder and more humid days. However, neither this finding (p-value = 0.115) nor other combinations of meteorological states reached a normative statistical significance to claim this association. This is most probably due to insufficient power, judging by relatively stable and consistent direction of point estimates of association between extreme conditions and vertigo. Waves of extreme humidity appeared to be associated with the onset of vertigo, albeit with borderline statistical significance. However, literature lacks a clear explanation of the underlying pathophysiological mechanisms related to changes in humidity.

The most notable and consistent finding in this analysis is the potential influence of elevated barometric pressure sustained for at least two consecutive days on the onset of vertigo (OR = 1.21, p = 0.024). This effect was particularly evident in patients with chronic hypertension (OR = 1.40, with a non-significant tendency p-value = 0.073).

There are two complementary physiological pathways that may underline these associations.

First, thermal and humidity-related mechanisms may play a role. Exposure to hotter or drier climatic conditions may disrupt the body’s systemic water and electrolyte balance, potentially prompting the inner ear to retain fluid through the secretion of natriuretic peptides, thereby triggering the onset of vertigo [19]. There are several ion channels and transporters expressed in both the inner ear and the kidney, playing critical roles in homeostasis and fluid balance. Although common physiological mechanisms linking these organs have not been fully established, it is plausible that they share regulatory pathways. The kidneys, as a central regulator of water and electrolyte balance, are highly sensitive to environmental conditions, particularly changes in weather. It is reasonable to hypothesize that physiological changes affecting renal ion transport due to weather variations could also impact ion channel function in the inner ear, potentially altering endolymph composition and increasing the risk of Vertigo episodes [20].

Second, barometric and vascular pathways may contribute. Elevated barometric pressure may directly affect endolymphatic balance in the inner ear, potentially leading to endolymphatic hydrops, which has been shown to be associated with vertigo and Ménière’s disease [21].

Hypertension-related vascular mechanisms may further amplify this effect. Sfakianaki et al. suggested that microvascular alterations and ischemic events associated with hypertension may contribute to otoconial detachment from the otolithic membrane, increasing the risk of BPPV recurrence. Given that the inner ear relies on terminal circulation, vascular insufficiency, especially when it affects the anterior inferior cerebellar artery (AICA) or vertebrobasilar artery (VBA), can result in audio-vestibular dysfunction. The vestibular system undergoes age-related degeneration, which is further exacerbated by hypertension and atherosclerosis, leading to progressive detachment of otoconia. Their work highlights that patients with hypertension and hyperlipidemia exhibit the highest recurrence rates of BPPV (55.89% and 67.80%, respectively), reinforcing the vascular hypothesis. While some studies did not find a statistically significant correlation, the cumulative evidence suggests that managing hypertension and metabolic disorders could help reduce BPPV recurrence [22]. Chen et al. showed that Hypertension plays a role as a risk factor to BPPV as well [23].

Medication-related influences may also play a role. As part of hypertension treatment, diuretics, such as thiazides, are commonly prescribed [24]. Since ion channels and transporters are shared between the inner ear and the kidney, it is possible that the impact of hypertension on vertigo incidence is partially mediated using antihypertensive medications. Studies suggest that thiazides, by altering fluid balance and electrolyte homeostasis, may reduce vestibular symptoms in hypertensive patients by mitigating endolymphatic hydrops, a condition linked to vertigo [25, 26]. While these drugs have demonstrated efficacy in lowering blood pressure and reducing cardiovascular risk, their impact on vertigo and BPPV recurrence remains to be fully elucidated. Further research is required to determine whether antihypertensive treatment, rather than hypertension itself, contributes to the increased prevalence of vestibular disorders in hypertensive patients.

Pressure-sensitivity of vestibular structures offers additional support for this interpretation.

Previous research reported an increased sensitivity of vestibular receptors to pressure and the valve-like structure that regulates endolymphatic pressure in the inner ear [27–29]. The dysfunction of this system prevents endolymphatic outflow and thus changes the regulation of inner ear pressure with fluctuation of atmospheric pressure [30]. Transmission of the pressure throughout the inner ear cavity may lead to dislodgement of otoconia leading to the symptoms of BPPV or Meniere’s Disease [11].

Limited information is available regarding the relationship between barometric pressure and blood pressure. From research of hyperbaric oxygen therapy, we know that high pressure and hyperoxygenation may cause peripheral vasoconstriction and increased systolic and mean arterial blood pressure [31]. It can be hypothesized that similar to the effects during hyperbaric oxygen therapy, in real-life conditions, elevation of barometric pressure may elevate systemic blood pressure, especially in hypertensive patients. This increase in blood pressure elevation combined with vertigo may worsen the patients’ symptoms.

The findings warrant increasing awareness for high daily barometric pressure levels and suggest primary care practitioners consider preventive recommendations for susceptible patients. In practice, such recommendations may include advising older adults or patients with balance disorders to exercise greater caution during high-pressure days, when vertigo episodes are more likely to occur. As these individuals are at increased risk of falls and injury, heightened awareness and proactive counseling could help reduce preventable harm.

Study has its limitations. For instance, the vertigo condition in the main analysis was classified as a general diagnosis based only on ICD-9, which did not distinguish between vertigo subtypes. This was done to minimize the selection bias. Using a general definition, not confirmed by ENT specialist, may potentially result in a misclassification bias. That said, the misclassification would likely reduce sample efficiency and bias the results toward the null hypothesis. However, many of the findings demonstrated statistically significant or near-significant results, leading us to believe that this limitation had only a minimal impact on our conclusions. In addition, Meniere’s disease was found to be extremely rare in our dataset (only 8 cases out of 3,429), which precluded meaningful subgroup analysis and should be considered a limitation when interpreting the generalizability of the findings to this condition.

The use of national rather than regional viral exposure data may have introduced exposure misclassification, limiting the precision of our estimates. Likewise, we would like to acknowledge the exploratory nature of our analyses, where we intentionally did not use multiple testing correction, in attempt to reveal all possible leads between vertigo to environment. This approach carries a risk of false-positive findings and should be noted particularly when interpreting any tendency suggesting findings.

The study benefited from being based on an entire population served by a single medical center, ensuring that no cases of vertigo were missed in the analysis.

## 5. Conclusion

The study findings highlight the importance of raising awareness about days with elevated barometric pressure levels, particularly among patients with hypertension or other vulnerable populations. Unlike common meteorological parameters such as temperature and humidity, barometric pressure levels are rarely reported but should be made accessible to the public.

Furthermore, primary care practitioners are encouraged to provide preventive recommendations to their at-risk patients based on these findings. Along with the findings observed for barometric pressure, our findings also suggested potential, though non-significant, trends related to temperature and humidity. Future research should explore these meteorological factors in larger datasets, across diverse geographical regions, to clarify their contribution to vertigo onset. It should also evaluate whether integrating meteorological monitoring with clinical data may help identify high-risk periods for vertigo onset.

## Supplementary information

**Additional file 1:** **Table S1:** ICD9 codes used for Vertigo definition. **Table S2.** Association between a wave of extremely high barometric pressure and vertigo onset. Results of subgroup analysis. **Figure S1**. Response curve between meteorological states and vertigo on the day of symptoms onset.

## Declarations

### Data availability

The datasets generated and analysed during the current study are not publicly available due to sensitivity of patients’ personal characteristics included in the analysis but can be made available upon receiving an additional approval by the IRB committee.
