# Supplementary material for: Environmental drivers of vertigo
Source: Environ Health Prev Med. 2026 Feb 17;31:10. doi: 10.1265/ehpm.25-00257 (PMC12950341; doi:10.1265/ehpm.25-00257)
Supplement: Supplementary file 1 — Additional file 1: Table S1: ICD9 codes used for Vertigo definition. Table S2. Association between a wave of extremely high barometric pressure and vertigo onset. Results of subgroup analysis. Figure S1. Response curve between meteorological states and vertigo on the day of symptoms onset. [file ehpm-31-010-s001.docx]

**Supplementary Materials**

**Table S1:** ICD9 codes used for Vertigo definition

| **Definition the manuscript** | **ICD9 code** | **Code description** |
| --- | --- | --- |
| Dizziness and giddiness (Vertigo) | 780.40 | Dizziness and giddiness (vertigo) |
| Vertigo, specific diagnosis | 43885 | Vertigo |
|  | 386.XX | Active meniere's disease, cochleovestibular |
|  |  | Active meniere's disease, vestibular |
|  |  | Benign paroxysmal positional vertigo |
|  |  | Labyrinthine dysfunction |
|  |  | Labyrinthitis |
|  |  | Labyrinthitis, unspecified |
|  |  | Meniere's disease |
|  |  | Meniere's disease, unspecified |
|  |  | Other and unspecified peripheral vertigo |
|  |  | Other peripheral vertigo |
|  |  | Peripheral vertigo, unspecified |
|  |  | Serous labyrinthitis |
|  |  | Unspecified vertiginous syndromes and labyrinthine disorders |
|  |  | Vertiginous syndromes and other disorders of vestibular system |
|  |  | Vertigo of central origin |
|  |  | Vestibular neuronitis |

**Table S2.** Association between a wave of extremely high barometric pressure and vertigo onset. Results of subgroup analysis.

| Subgroup | OR^1^ (95%CI) | p-value |
| --- | --- | --- |
| Age group  18-45  45-65  65+ | 1.22 (0.89; 1.67)  1.24 (0.96; 1.60)  1.17 (0.85; 1.60) | 0.227  0.095  0.334 |
| Sex  Females  Males | 1.24 (1.00; 1.54)  1.17 (0.90; 1.52) | 0.055  0.236 |
| Ethnicity  Bedouin-Arab  Jewish | 1.18 (0.80; 1.72)  1.22 (1.01; 1.47) | 0.404  0.035 |
| Chronic hypertension  No  Yes | 1.17 (0.97; 1.41)  1.40 (0.97; 2.02) | 0.101  0.073 |
| Diabetes Mellitus  No  Yes | 1.24 (1.03; 1.49)  1.07 (0.71; 1.63) | 0.020  0.743 |
| Fever  ≤37^0^C  >37^0^C | 1.27 (1.05; 1.54)  1.08 (0.63; 1.84) | 0.016  0.778 |

^1^ All effect estimates are adjusted to temperature and RH on the day of the event onset or its control.

**Figure S1**. Response curve between meteorological states and vertigo on the day of symptoms onset

| *Temperature* | *Relative humidity* | *Barometric pressure* |
| --- | --- | --- |
| 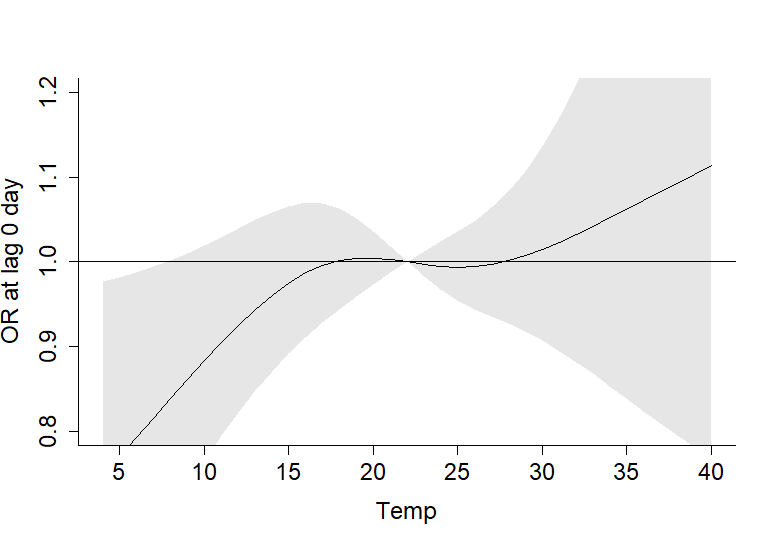 | 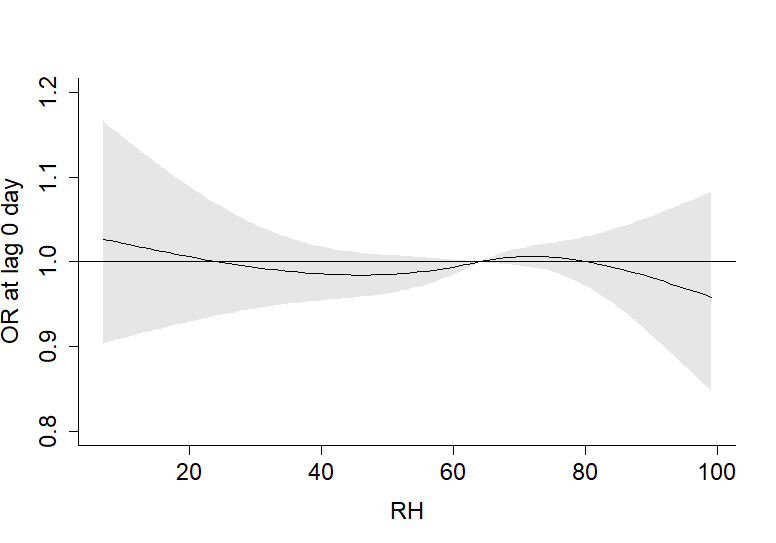 | 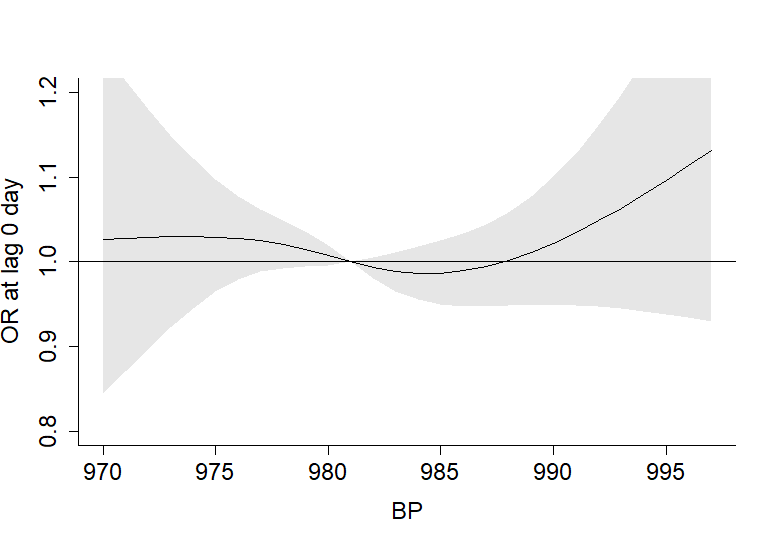 |
| *An effect of cumulative exposure to* ***10^th^*** *percentile vs median for a week prior to onset OR(95%CI)* | | |
| 1.03 (0.81; 1.32) | 0.99 (0.89; 1.10) | 1.04 (0.91; 1.19) |
| *An effect of cumulative exposure to* ***90^th^*** *percentile vs median for a week prior to onset*  *OR (95%CI)* | | |
| 1.20 (0.96; 1.50) | 0.92 (0.81; 1.05) | 0.94 (0.82; 1.08) |

*Risk estimates of temperature, relative humidity and barometric pressure are adjusted to each other, and centered around their respective global median.
